# Genome Sequence of a *Bacillus anthracis* Outbreak Strain from Zambia, 2011

**DOI:** 10.1128/genomeA.00116-14

**Published:** 2014-03-06

**Authors:** Naomi Ohnishi, Fumito Maruyama, Hirohito Ogawa, Hirokazu Kachi, Shunsuke Yamada, Daisuke Fujikura, Ichiro Nakagawa, Mudenda B. Hang’ombe, Yuka Thomas, Aaron S. Mweene, Hideaki Higashi

**Affiliations:** a Division of Infection and Immunity, Research Center for Zoonosis Control, Hokkaido University, Sapporo, Japan; b Section of Microbial Genomics and Ecology, Tokyo Medical and Dental University, Tokyo, Japan; c Hokudai Center for Zoonosis Control in Zambia, Hokkaido University, Lusaka, Zambia; d Section of Bacterial Pathogenesis, Graduate School of Medical and Dental Sciences, Tokyo Medical and Dental University, Tokyo, Japan; e School of Veterinary Medicine, University of Zambia, Lusaka, Zambia

## Abstract

In August 2011, an anthrax outbreak occurred among *Hippopotamus amphibius* hippopotamuses and humans in Zambia. Here, we report the draft genome sequence of the *Bacillus anthracis* outbreak strain CZC5, isolated from tissues of *H. amphibius* hippopotamuses that had died in the outbreak area.

## GENOME ANNOUNCEMENT

Anthrax is a life-threatening disease caused by the Gram-positive spore-forming bacterium *Bacillus anthracis* (1). The disease occurs worldwide, and outbreaks and epidemics of the disease in both humans and animals have been reported in Zambia (2, 3). In August 2011, an anthrax outbreak occurred among *Hippopotamus amphibius* hippopotamuses in the Chama district of the eastern province of Zambia (4). Over 80 hippopotamuses died after showing signs of infection with *B. anthracis*. Following the deaths of the hippopotamuses, 521 suspected human cases resulting in 6 deaths were reported. As of January 2014, 7 completed genome sequences and 23 draft genome sequences for *B. anthracis* had been deposited in GenBank. However, most of these belong to strains that were isolated in the United States; there have been no reports on the genome sequences of *B. anthracis* strains isolated in central Africa. Here, we report the draft genome sequence of a *B. anthracis* strain associated with an outbreak linked to unnatural deaths of hippopotamuses in Zambia in 2011.

The genomic DNA was extracted from overnight cultures using a Qiagen DNeasy blood and tissue kit, according to the supplied protocol. A library for sequencing was prepared from the extracted genomic DNA using a Nextera XT sample preparation kit (Illumina), and paired-end sequencing with a read length of 2 × 300 bp was performed on an Illumina MiSeq platform. The total amount of read data comprises 1,223,422,928 bases, representing 234-fold coverage of the genome. After obtaining reads that passed the quality threshold with the Trimmomatic-0.20 tool (5) and merging overlapping paired-end reads with FastqJoin (https://code.google.com/p/ea-utils/), a mapping assembly was carried out with Newbler version 2.9 (Roche). The assembled genome consists of 25 contigs of >301 bp (N_50_ contig size, 195,537 bp), with an average G+C content of 35.12%. The draft genome was structurally and functionally annotated using the Rapid Annotations using Subsystems Technology (RAST) annotation server (6). A total of 5,841 putative coding sequences (CDS), 11 rRNA operons, and 91 tRNA genes were identified. The quality-passed reads were aligned to the reference *B. anthracis* strain Ames Ancestor (taxonomy ID 261594) using SSAHA2 (7) as part of the Breseq pipeline version 0.24rc4 (8), resulting in only 1 missing gene (that for aspartate ammonia-lyase [*aspA-3*]) in this strain.

We believe that a detailed comparative genomic analysis of this *B. anthracis* strain, designated CZC5, with more genome sequences of this species obtained in Zambia might lead to new insights into its evolutionary history and pathogenicity. The results of such studies combined with data for worldwide lineages will be included in a future publication.

### Nucleotide sequence accession number.

This whole-genome shotgun project for *B. anthracis* strain CZC5 has been deposited at DDBJ/ENA/GenBank under the accession no. BAVT00000000. The version described in this paper is the first version.

## References

[1] Dixon TC Meselson M Guillemin J Hanna PC 1999 Anthrax. N. Engl. J. Med. 341:815–826. 10.1056/NEJM199909093411107 10477781

[2] Siamudaala VM Bwalya JM Munang’andu HM Sinyangwe PG Banda F Mweene AS Takada A Kida H Kida H 2006 Ecology and epidemiology of anthrax in cattle and humans in Zambia. Jpn. J. Vet. Res. 54:15–23 16786974

[3] Tuchili LM Pandey GS Sinyangwe PG Kaji T 1993 Anthrax in cattle, wildlife and humans in Zambia. Vet. Rec. 132:487. 10.1136/vr.132.19.487-a 8506601

[4] Hang’ombe MB Mwansa JC Muwowo S Mulenga P Kapina M Musenga E Squarre D Mataa L Thomas SY Ogawa H Sawa H Higashi H 2012 Human-animal anthrax outbreak in the Luangwa valley of Zambia in 2011. Trop. Doct. 42:136–139. 10.1258/td.2012.110454 22472314

[5] Lohse M Bolger AM Nagel A Femie AR Lunn JE Stitt M Usadel B 2012 RobiNA: a user-friendly, integrated software solution for RNA-Seq-based transcriptomics. Nucleic Acids Res. 40:W622–W627. 10.1093/nar/gks540 22684630 PMC3394330

[6] Aziz RK Bartels D Best AA DeJongh M Disz T Edwards RA Formsma K Gerdes S Glass EM Kubal M Meyer F Olsen GJ Olson R Osterman AL Overbeek RA McNeil LK Paarmann D Paczian T Parrello B Pusch GD Reich C Stevens R Vassieva O Vonstein V Wilke A Zagnitko O 2008 The RAST server: Rapid Annotations using Subsystems Technology. BMC Genomics 9:75. 10.1186/1471-2164-9-75 18261238 PMC2265698

[7] Ning Z Cox AJ Mullikin JC 2001 SSAHA: a fast search method for large DNA databases. Genome Res. 11:1725–1729. 10.1101/gr.194201 11591649 PMC311141

[8] Barrick JE Yu DS Yoon SH Jeong H Oh TK Schneider D Lenski RE Kim JF 2009 Genome evolution and adaptation in a long-term experiment with *Escherichia coli*. Nature 461:1243–1247. 10.1038/nature08480 19838166

